# Supplementary material for: Disentangling signal and noise in neural responses through generative modeling
Source: bioRxiv. 2024 Aug 22:2024.04.22.590510. Originally published 2024 Apr 27. Preprint. [Version 2] doi: 10.1101/2024.04.22.590510 (PMC11071385; doi:10.1101/2024.04.22.590510)
Supplement: Supplement 1 [file NIHPP2024.04.22.590510v2-supplement-1.pdf]

bioRxiv preprint doi: https://doi.org/10.1101/2024.04.22.590510; this version posted August 22, 2024. The copyright holder for this preprint (which was not certified by peer review) is the author/funder, who has granted bioRxiv a license to display the preprint in perpetuity. It is made available under aCC-BY 4.0 International license.

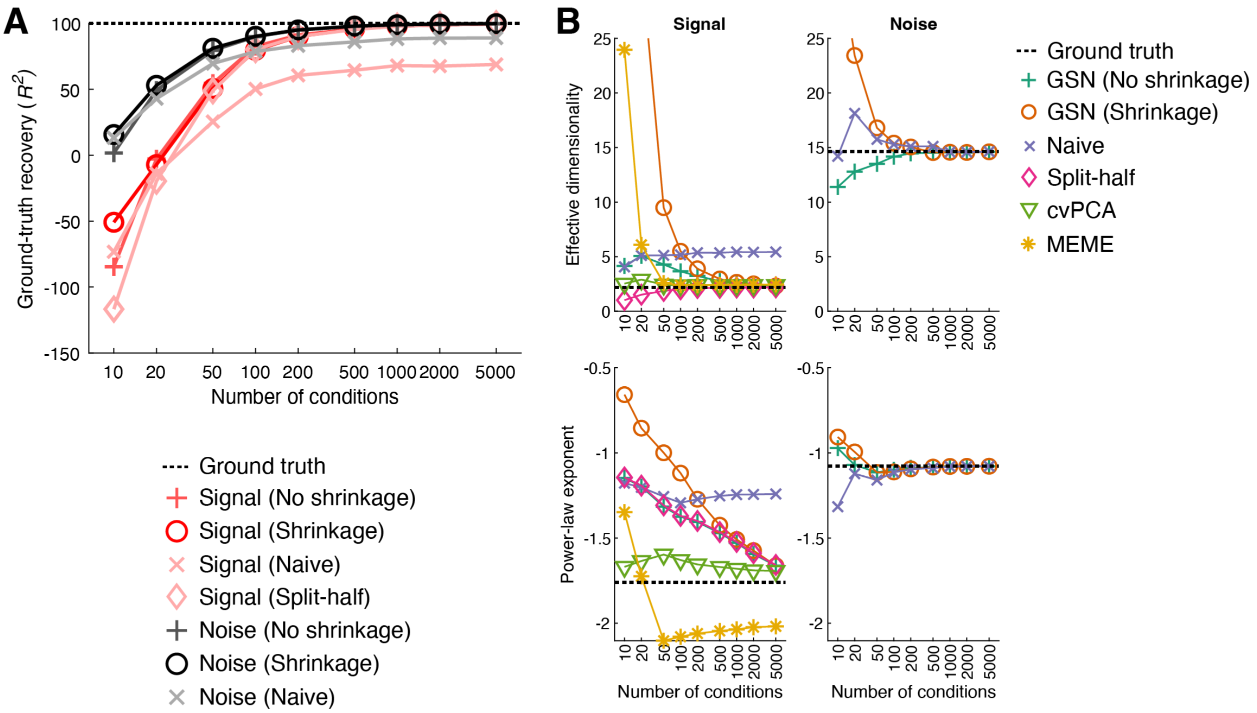

S1 Figure. Simulations for empirically derived signal and noise covariance. Here, we show simulation results for a scenario in which we take the ground-truth signal and noise covariance to be the GSN estimates of signal and noise covariance obtained from FFA-1 as illustrated in **Figure 6** (code available at https://osf.io/3yvtg). *A*, Same format as **Figure 4C**. Results are similar to those found in **Figure 4C**. *B*, Same format as **Figure 5**. The results for effective dimensionality (ED) look similar to those found in **Figure 5**. However, the results for power-law exponent look different. Specifically, the methods exhibit poor recovery of signal power-law exponent: each method either takes a very large amount of data to converge towards ground truth or has biases that do not resolve with additional data. One potential explanation is that the ground-truth signal covariance in this scenario is not exactly a line in log-log space (i.e. a power-law function), whereas all of the scenarios shown in **Figure 5** are exactly linear in log-log space. Hence, recovery may be especially difficult to achieve for the current scenario. Arguably, ED is a more appropriate metric for the evaluation of methods here, as it makes minimal assumptions about the structure of the eigenspectrum. Also, note that for sake of consistency with the other simulations, the MEME method was run assuming an unbroken power-law function; in theory, the MEME method could be run assuming a broken power-law function, which might help better match the ground-truth signal eigenspectrum and improve results.

bioRxiv preprint doi: https://doi.org/10.1101/2024.04.22.590510; this version posted August 22, 2024. The copyright holder for this preprint (which was not certified by peer review) is the author/funder, who has granted bioRxiv a license to display the preprint in perpetuity. It is made available under aCC-BY 4.0 International license.

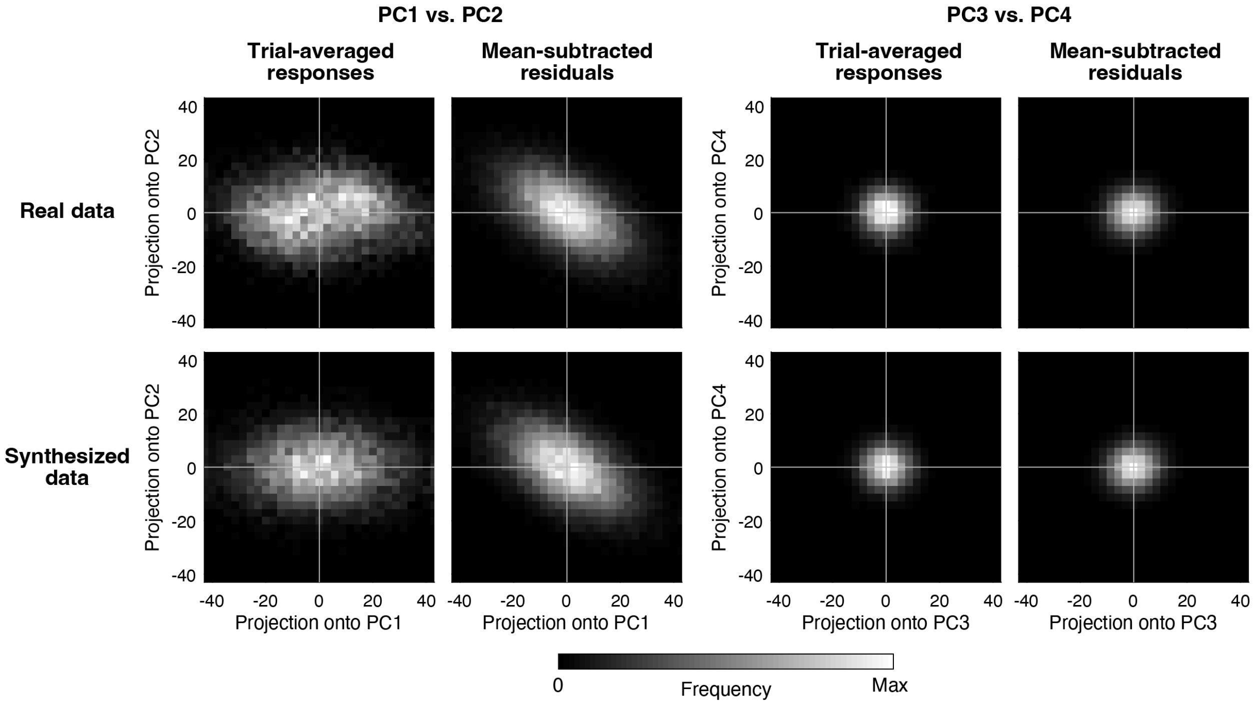

S2 Figure. Assessment of data distributions. As an instructive exercise, we take the empirical brain data from FFA-1 illustrated in **Figure 6** and perform an inspection of the signal and noise components of the data (code available at https://osf.io/yxrsp). We inspect two different distributions. One is the distribution of trial-averaged responses. Since trial averaging reduces noise, inspecting trial-averaged responses helps us assess properties of the signal. The second is the distribution of mean-subtracted residuals (in which trial-averaged responses have been removed). This allows us to focus our assessment on properties of the noise. We compute the first several principal components (PCs) of the covariance of the trial-averaged responses and then visualize the two distributions of interest in the low-dimensional space defined by these PCs. We also generate, for comparison, a synthesized dataset based on the parameters of the GSN model as fit to the empirical data. In order to generate responses for this synthesized dataset, we assume that both the signal and noise are Gaussian-distributed. We visualize the synthesized data in exactly the same manner as the real data (including using the same low-dimensional space). Examining the distributions associated with the real data (top row), we see that both the distribution of trial-averaged responses and the distribution of mean-subtracted residuals are Gaussian-like in their shape. We also see that the structure of the mean-subtracted residuals differs from that of the trial-averaged responses (top row, compare first and second images). This indicates that the noise structure is not identical to the signal structure, consistent with the inspections in **Figure 6A**. Next, we compare the distributions associated with the real data (top row) with those obtained from the synthesized data (bottom row). The distributions obtained from the synthesized data look very similar to those from the real data, suggesting that both the signal and the noise in the real data have Gaussian-like distributions and that the generative model learned by GSN accurately characterizes the real data.

bioRxiv preprint doi: https://doi.org/10.1101/2024.04.22.590510; this version posted August 22, 2024. The copyright holder for this preprint (which was not certified by peer review) is the author/funder, who has granted bioRxiv a license to display the preprint in perpetuity. It is made available under aCC-BY 4.0 International license.

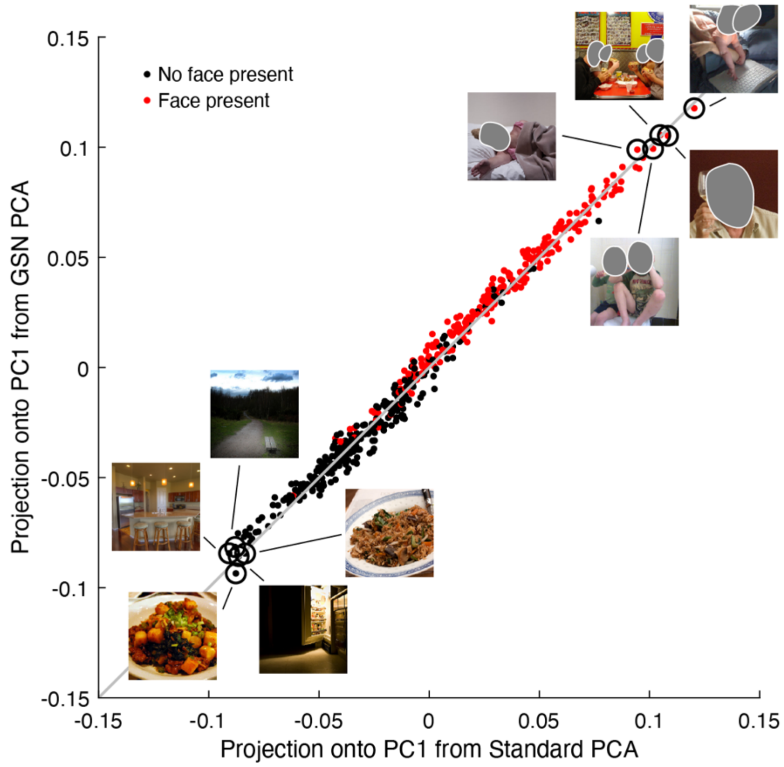

S3 Figure. Inspection of stimuli in PCA results. Here we visually inspect stimulus images from the PCA analysis (code available at https://osf.io/f34bc). The projections of responses in FFA-1 to the common 515 images onto PC1 (see **Figure 7C**) were unit-length-normalized, averaged across participants, and then unit-length-normalized again. This figure compares the results obtained using Standard PCA (*x*-axis) against the results obtained using GSN PCA (*y*-axis). Red dots indicate images that were judged by human raters to have at least one prominent face present; black dots indicate all other images. (The human raters were blind to the results in this paper.) The actual images corresponding to the highest five and lowest five projection values (based on the average of the results of the two methods) are shown. The presence of faces appears to be the dominant factor governing the response projections. (Note: Faces have been grayed out due to privacy reasons.)

bioRxiv preprint doi: https://doi.org/10.1101/2024.04.22.590510; this version posted August 22, 2024. The copyright holder for this preprint (which was not certified by peer review) is the author/funder, who has granted bioRxiv a license to display the preprint in perpetuity. It is made available under aCC-BY 4.0 International license.

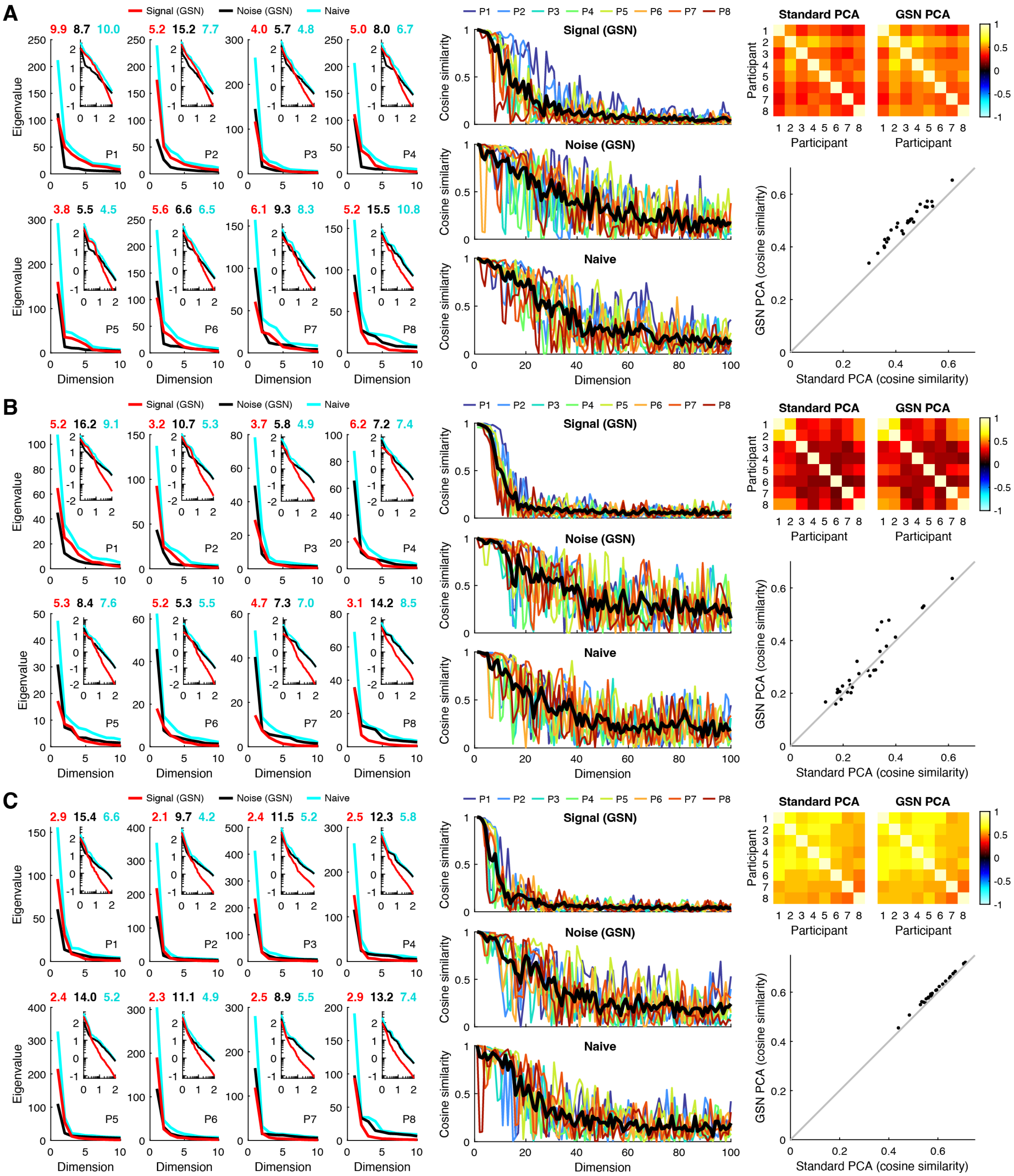

S4 Figure. PCA results for additional brain regions. Here we show results of the PCA analysis for additional brain regions (code available at https://osf.io/f34bc). The format is the same as used in **Figure 7**. *A–C*, Results for V1, hV4, and PPA, respectively. The main findings observed for FFA-1 in **Figure 7** replicate for these additional regions, including lower dimensionality for the signal compared to the noise, high within-participant reliability of the first several signal PCs and noise PCs, and higher across-participant consistency of trial-averaged response projections onto PC1 for GSN PCA than for standard PCA. Compared to FFA-1, the increase in across-participant consistency is more variable in hV4 and is relatively

bioRxiv preprint doi: https://doi.org/10.1101/2024.04.22.590510; this version posted August 22, 2024. The copyright holder for this preprint (which was not certified by peer review) is the author/funder, who has granted bioRxiv a license to display the preprint in perpetuity. It is made available under aCC-BY 4.0 International license.

small (but reliable) in PPA. One possible source of these region-wise differences may be differences in the degree to which signal covariance structure and noise covariance structure are aligned in different brain regions. For example, if noise covariance tends to align with signal covariance, then noise may have less of a corrupting influence on the estimation of signal PCs compared to when noise covariance is orthogonal to signal covariance.

bioRxiv preprint doi: https://doi.org/10.1101/2024.04.22.590510; this version posted August 22, 2024. The copyright holder for this preprint (which was not certified by peer review) is the author/funder, who has granted bioRxiv a license to display the preprint in perpetuity. It is made available under aCC-BY 4.0 International license.

S5 Appendix: GSN estimation of signal and noise covariance

Problem setting

As described in the main text, GSN calculates two covariance estimates from the data: $\hat{\Sigma}_{noiseORIG}$ and $\hat{\Sigma}_{data[t]}$. The former is an estimate of the noise covariance based on the trial-to-trial variability around the mean response to each condition (see Step 2). The latter is an estimate of the data covariance based on the data after averaging across t trials (see Step 3).

These two covariance estimates reflect unknown covariance matrices Σ_{signal} and Σ_{noise} such that $\hat{\Sigma}_{noiseORIG}$ is a noisy version of Σ_{noise} based on $c(t-1)$ samples and $\hat{\Sigma}_{data[t]}$ is a noisy version of $\Sigma_{data[t]} = \Sigma_{signal} + \Sigma_{noise}/t$ based on $c-1$ samples.

We wish to determine estimates $\hat{\Sigma}_{signal}$ and $\hat{\Sigma}_{noise}$ under the constraint that these estimates are positive semi-definite matrices. To do so, we define the following loss that quantifies errors from the data-derived covariances scaled by the number of samples they are based on:

$$L\left(\hat{\Sigma}_{signal}, \hat{\Sigma}_{noise}\right) = c(t-1)\left\|\hat{\Sigma}_{noiseORIG} - \hat{\Sigma}_{noise}\right\|_2^2 + (c-1)\left\|\left(\hat{\Sigma}_{data[t]} - \hat{\Sigma}_{noise}/t\right) - \hat{\Sigma}_{signal}\right\|_2^2$$

where $\| \|_2$ indicates the Frobenius norm. Intuitively, the noise estimate $\hat{\Sigma}_{noise}$ is allowed to deviate to some degree from the data-derived $\hat{\Sigma}_{noiseORIG}$, and the signal estimate $\hat{\Sigma}_{signal}$ is allowed to deviate to some degree from the subtraction-based estimate of the signal covariance $\hat{\Sigma}_{data[t]} - \hat{\Sigma}_{noise}/t$.

Notice $\hat{\Sigma}_{noiseORIG}$ is positive semi-definite, as it is a covariance matrix computed from data. If $\hat{\Sigma}_{data[t]} - \hat{\Sigma}_{noise}/t$ is also positive semi-definite, setting $\hat{\Sigma}_{noise} = \hat{\Sigma}_{noiseORIG}$ and $\hat{\Sigma}_{signal} = \hat{\Sigma}_{data[t]} - \hat{\Sigma}_{noise}/t$ is the optimal solution for the problem (since the loss equals zero). If not, we can solve the optimization problem using the method described below.

Solution

To solve the optimization problem in the general case, we note that it is a convex optimization problem, as it is a sum of squares and the cone of semi-definite matrices is a convex set (Boyd and Vandenberghe, 2016). Thus, this problem has a single optimum. For solving this problem efficiently, we split the problem into optimizing $\hat{\Sigma}_{signal}$ and $\hat{\Sigma}_{noise}$ separately, as we can compute an analytic solution for each matrix if the other is fixed. Since each of these separate optimizations is guaranteed to improve the loss, this approach is guaranteed to converge.

Lemma: solution pattern

Consider the following problem. Given B, find A that minimizes $\|B-A\|_2^2$ (or equivalently $\|A-B\|_2^2$) subject to the constraint that A is positive semi-definite. We can solve this problem as $A = \mathrm{PSD}(B)$ where $\mathrm{PSD}()$ is the method for finding the nearest positive semi-definite matrix described in the main text. We will use this solution pattern in solving the individual optimizations for $\hat{\Sigma}_{signal}$ and $\hat{\Sigma}_{noise}$.

Optimizing $\hat{\Sigma}_{signal}$

Since $c(t-1)\left\|\hat{\Sigma}_{noiseORIG} - \hat{\Sigma}_{noise}\right\|_2^2$ is independent of $\hat{\Sigma}_{signal}$, we are left with minimizing

bioRxiv preprint doi: https://doi.org/10.1101/2024.04.22.590510; this version posted August 22, 2024. The copyright holder for this preprint (which was not certified by peer review) is the author/funder, who has granted bioRxiv a license to display the preprint in perpetuity. It is made available under aCC-BY 4.0 International license.

$$(c-1)\left\|\left(\hat{\Sigma}_{data[t]}-\hat{\Sigma}_{noise}/t\right)-\hat{\Sigma}_{signal}\right\|_2^2$$

subject to $\hat{\Sigma}_{signal}$ being positive semi-definite. To do this, we use our solution pattern where $A=\hat{\Sigma}_{signal}$ and $B=\hat{\Sigma}_{data[t]}-\hat{\Sigma}_{noise}/t$.

Optimizing $\hat{\Sigma}_{noise}$

In this case, we apply a quadratic extension to turn the sum of squares into a single one:

$$L\left(\hat{\Sigma}_{signal},\hat{\Sigma}_{noise}\right)=\sum_{ij}\left[c(t-1)\left(\hat{\Sigma}^{(ij)}_{noiseORIG}-\hat{\Sigma}^{(ij)}_{noise}\right)^2+(c-1)\left(\hat{\Sigma}^{(ij)}_{data[t]}-\hat{\Sigma}^{(ij)}_{signal}-\frac{\hat{\Sigma}^{(ij)}_{noise}}{t}\right)^2\right]$$

$$=\sum_{ij}\left[c(t-1)\left(\left(\hat{\Sigma}^{(ij)}_{noiseORIG}\right)^2-2\hat{\Sigma}^{(ij)}_{noiseORIG}\hat{\Sigma}^{(ij)}_{noise}+\left(\hat{\Sigma}^{(ij)}_{noise}\right)^2\right)\right.$$

$$\left.+\frac{c-1}{t^2}\left(\left(t^2\left(\hat{\Sigma}^{(ij)}_{data[t]}-\hat{\Sigma}^{(ij)}_{signal}\right)^2\right)-2t\left(\hat{\Sigma}^{(ij)}_{data[t]}-\hat{\Sigma}^{(ij)}_{signal}\right)\hat{\Sigma}^{(ij)}_{noise}+\left(\hat{\Sigma}^{(ij)}_{noise}\right)^2\right)\right]$$

$$=\sum_{ij}\left[\frac{ct^2(t-1)+c-1}{t^2}\left(\hat{\Sigma}^{(ij)}_{noise}\right)^2-2\hat{\Sigma}^{(ij)}_{noise}\left(c(t-1)\hat{\Sigma}^{(ij)}_{noiseORIG}+\frac{c-1}{t}\left(\hat{\Sigma}^{(ij)}_{data[t]}-\hat{\Sigma}^{(ij)}_{signal}\right)\right)\right]+C_0$$

where C_0 is a term that is independent of $\hat{\Sigma}_{noise}$. Simplifying, we obtain:

$$L\left(\hat{\Sigma}_{signal},\hat{\Sigma}_{noise}\right)\propto\sum_{ij}\left[\hat{\Sigma}^{(ij)}_{noise}-\frac{ct^2(t-1)}{ct^2(t-1)+c-1}\hat{\Sigma}^{(ij)}_{noiseORIG}-\frac{c-1}{ct^2(t-1)+c-1}t\left(\hat{\Sigma}^{(ij)}_{data[t]}-\hat{\Sigma}^{(ij)}_{signal}\right)\right]^2+C_1$$

where C_1 is a term that is independent of $\hat{\Sigma}_{noise}$. To minimize this loss, we use our solution pattern where $A=\hat{\Sigma}_{noise}$ and $B=\frac{ct^2(t-1)}{ct^2(t-1)+c-1}\hat{\Sigma}_{noiseORIG}+\frac{c-1}{ct^2(t-1)+c-1}t\left(\hat{\Sigma}_{data[t]}-\hat{\Sigma}_{signal}\right)$.

Notice that the calculation of B is a weighted average of two possible estimates of the noise covariance. The first estimate, $\hat{\Sigma}_{noiseORIG}$, reflects the covariance of mean-subtracted residuals, while the second estimate, $t\left(\hat{\Sigma}_{data[t]}-\hat{\Sigma}_{signal}\right)$, reflects the subtraction of the signal distribution from the data distribution. The weights in the weighted average reflect the amount of data that inform each of the two estimates.

Algorithm

The overall algorithm for optimizing signal and noise covariance estimates is described in the main text. Holding $\hat{\Sigma}_{noise}$ fixed, the algorithm optimizes $\hat{\Sigma}_{signal}$ in Step 6.1. Holding $\hat{\Sigma}_{signal}$ fixed, the algorithm optimizes $\hat{\Sigma}_{noise}$ in Step 6.2. This process of biconvex optimization is iterated until convergence.

Proof that projection reduces error

We claim in the main text that projection of a given covariance estimate onto the positive semi-definite cone always reduces the error of the estimate. Here we provide a simple proof of this claim.

Definitions: For this proof, let Σ be the true *n*-dimensional covariance which lies within the convex cone of positive semi-definite matrices $C\subset\mathbb{R}^{n\times n}$. We assume the original covariance estimate $\hat{\Sigma}\notin C$.

Theorem: Under these conditions, the squared error of the projection onto the positive semi-definite cone $\mathrm{PSD}(\hat{\Sigma})$ is smaller than the squared error of the original estimate, i.e.:

$$\left\|\Sigma-\mathrm{PSD}(\hat{\Sigma})\right\|_2^2<\left\|\Sigma-\hat{\Sigma}\right\|_2^2$$

bioRxiv preprint doi: https://doi.org/10.1101/2024.04.22.590510; this version posted August 22, 2024. The copyright holder for this preprint (which was not certified by peer review) is the author/funder, who has granted bioRxiv a license to display the preprint in perpetuity. It is made available under aCC-BY 4.0 International license.

Proof: As C is convex, there is a tangent plane touching C at $\mathrm{PSD}(\hat{\Sigma})$ to which the vector from $\hat{\Sigma}$ to $\mathrm{PSD}(\hat{\Sigma})$ is orthogonal. All points in C are on the other side of this tangent plane compared to $\hat{\Sigma}$. The squared distance from $\hat{\Sigma}$ to Σ can be decomposed into the distance orthogonal to the tangent plane and the distance within the tangent plane. The distance within the plane is the same for $\hat{\Sigma}$ and $\mathrm{PSD}(\hat{\Sigma})$, and the distance orthogonal to the plane is smaller for $\mathrm{PSD}(\hat{\Sigma})$. Thus, the total distance for $\mathrm{PSD}(\hat{\Sigma})$ is indeed smaller than the total distance for $\hat{\Sigma}$. See **Figure S5.1** for a helpful illustration.

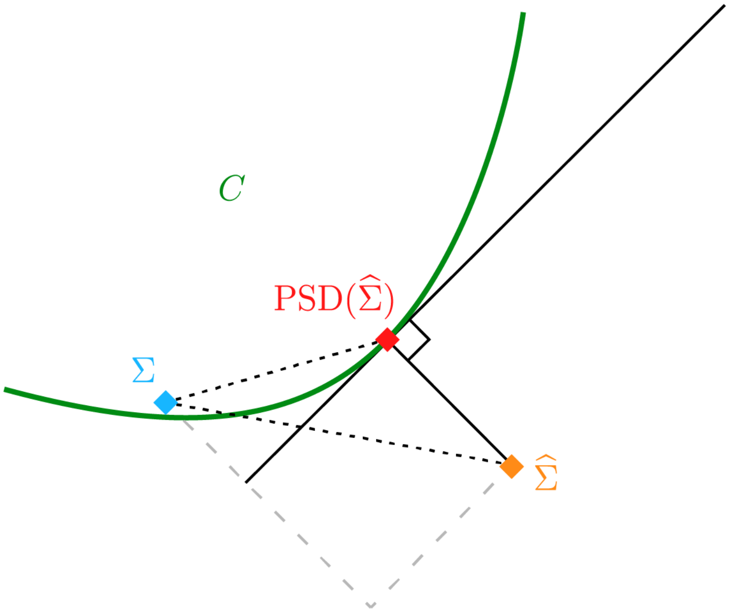

Figure S5.1. Illustration that projection onto the positive semi-definite cone reduces error.

Rationale for squared error

Our estimates are based on minimizing sum of squares, i.e., we minimize the squared difference between our estimates and the data-derived $\hat{\Sigma}_{noiseORIG}$ and $\hat{\Sigma}_{data[t]}$. Squared error is a common loss for the estimation of covariance matrices, and in particular, it is the loss optimized by the shrinkage method we employ for covariance estimation. Additionally, squared error is a convex loss function, which guarantees that our fitting procedure converges.

We note that our squared-error loss does not correspond to a log likelihood under some distributional assumption. Rather, it is merely a mathematically convenient way to express the trade-off between the two data-driven covariance estimates $\hat{\Sigma}_{noiseORIG}$ and $\hat{\Sigma}_{data[t]}$. Typical likelihood functions for covariance matrices imply larger variabilities for larger entries in the covariance matrix, but this is not the case for our squared-error loss.

In our squared-error loss, we weight the two errors (one for $\hat{\Sigma}_{noiseORIG}$, one for $\hat{\Sigma}_{data[t]}$) by the relevant degrees of freedom. This is a sensible approach that adapts to the specific numbers of conditions and trials used in a given experiment. We acknowledge that it may be possible to devise a more principled approach for determining the weighting. Nonetheless, note that the relative weighting of the errors does not change

bioRxiv preprint doi: https://doi.org/10.1101/2024.04.22.590510; this version posted August 22, 2024. The copyright holder for this preprint (which was not certified by peer review) is the author/funder, who has granted bioRxiv a license to display the preprint in perpetuity. It is made available under aCC-BY 4.0 International license.

fundamental properties of the estimators. For any chosen weighting, $\hat{\Sigma}_{noise}$ and $\hat{\Sigma}_{signal}$ are positive semi-definite and approximate the data-derived covariance estimates.

References

Boyd, S., Vandenberghe, L., 2016. Convex Optimization. Cambridge University Press, Cambridge, England.

bioRxiv preprint doi: https://doi.org/10.1101/2024.04.22.590510; this version posted August 22, 2024. The copyright holder for this preprint (which was not certified by peer review) is the author/funder, who has granted bioRxiv a license to display the preprint in perpetuity. It is made available under aCC-BY 4.0 International license.

S6 Appendix: Shrinkage-based covariance estimation

A core component of GSN is estimation of covariance (this is performed for the estimated noise covariance in Step 2 and the estimated data covariance in Step 3). However, in high-dimensional datasets involving a large number of units but only a limited number of samples (e.g. trials), the standard method of computing sample covariance may yield inaccurate estimates of covariance. To improve accuracy of covariance estimation, GSN incorporates shrinkage (Ledoit and Wolf, 2004; Schäfer and Strimmer, 2005) of off-diagonal elements of covariance matrices towards zero. This reflects the prior that units are generally expected to be uncorrelated. The specific amount of shrinkage is tailored to optimally match the data using a cross-validation procedure in which likelihoods are evaluated on held-out data (see *Methods*).

We tested our shrinkage-based method for covariance estimation. We performed a set of simulations in which we assessed, as a function of the number of samples, how well the shrinkage method recovers a ground-truth covariance, compared to the standard method in which shrinkage is omitted (**Figure S6.1**). In one set of simulations, we used a ground-truth covariance equal to the identity matrix, corresponding to a scenario of uncorrelated units (panel A). In a second set of simulations, we re-used the previous ground-truth covariance but introduced positive correlations (r = 0.5) amongst the first five units (panel B). For additional comparison, in a third set of simulations, we used a ground-truth covariance equal to the covariance of a fixed set of random numbers drawn from the standard normal distribution (20 observations, 10 variables) (panel C).

The results show that the shrinkage method works well. In each scenario, the introduction of shrinkage improves ground-truth covariance recovery and this occurs regardless of the number of samples (panels A–C, lower left). Note that the size of the improvement varies across scenarios, with larger improvements when the ground truth is consistent with the prior of uncorrelated units (e.g. panel A) than when this is less the case (e.g. panel C). This makes sense: the cross-validation procedure should, in theory, correctly determine that shrinkage should be applied more strongly in situations where the underlying ground-truth covariance involves uncorrelated variables. Indeed, if we examine cross-validation results across different shrinkage levels, we see that in the scenario of uncorrelated variables, the shrinkage fractions yielding the highest likelihood on held-out data are close to 0, indicating large amounts of shrinkage (panel A, vertical red line in rightmost column), whereas in scenarios of correlated variables, the optimal shrinkage levels are closer to 1, indicating small amounts of shrinkage (panels B–C, vertical red lines in rightmost column).

The simulations also reveal insights into how ground-truth recovery performance varies as a function of the amount of data. As the number of samples increases, the induced shrinkage becomes weaker (compare 5 samples to 100 samples in panel C). This makes sense because at small sample sizes, the unregularized (non-shrunken) covariance is so inaccurate that inducing heavy bias improves the estimate. Furthermore, we see that as the number of samples increases, the difference in results between the shrinkage method and the standard method becomes smaller. Thus, shrinkage provides the most benefit when the amount of available data is small. It is important to keep in mind, however, that the covariance estimates produced by shrinkage are by no means perfect and that they contain bias. This can be seen intuitively by visually comparing the shrinkage-based covariance estimates at low number of samples to the ground-truth covariance. While shrinkage increases the overall similarity of covariance estimates to the underlying ground-truth covariance, it does so at the expense of biasing the magnitudes of off-diagonal elements towards zero. The introduction of bias is not necessarily a problem per se, as it depends on the goals of the researcher.

bioRxiv preprint doi: https://doi.org/10.1101/2024.04.22.590510; this version posted August 22, 2024. The copyright holder for this preprint (which was not certified by peer review) is the author/funder, who has granted bioRxiv a license to display the preprint in perpetuity. It is made available under aCC-BY 4.0 International license.

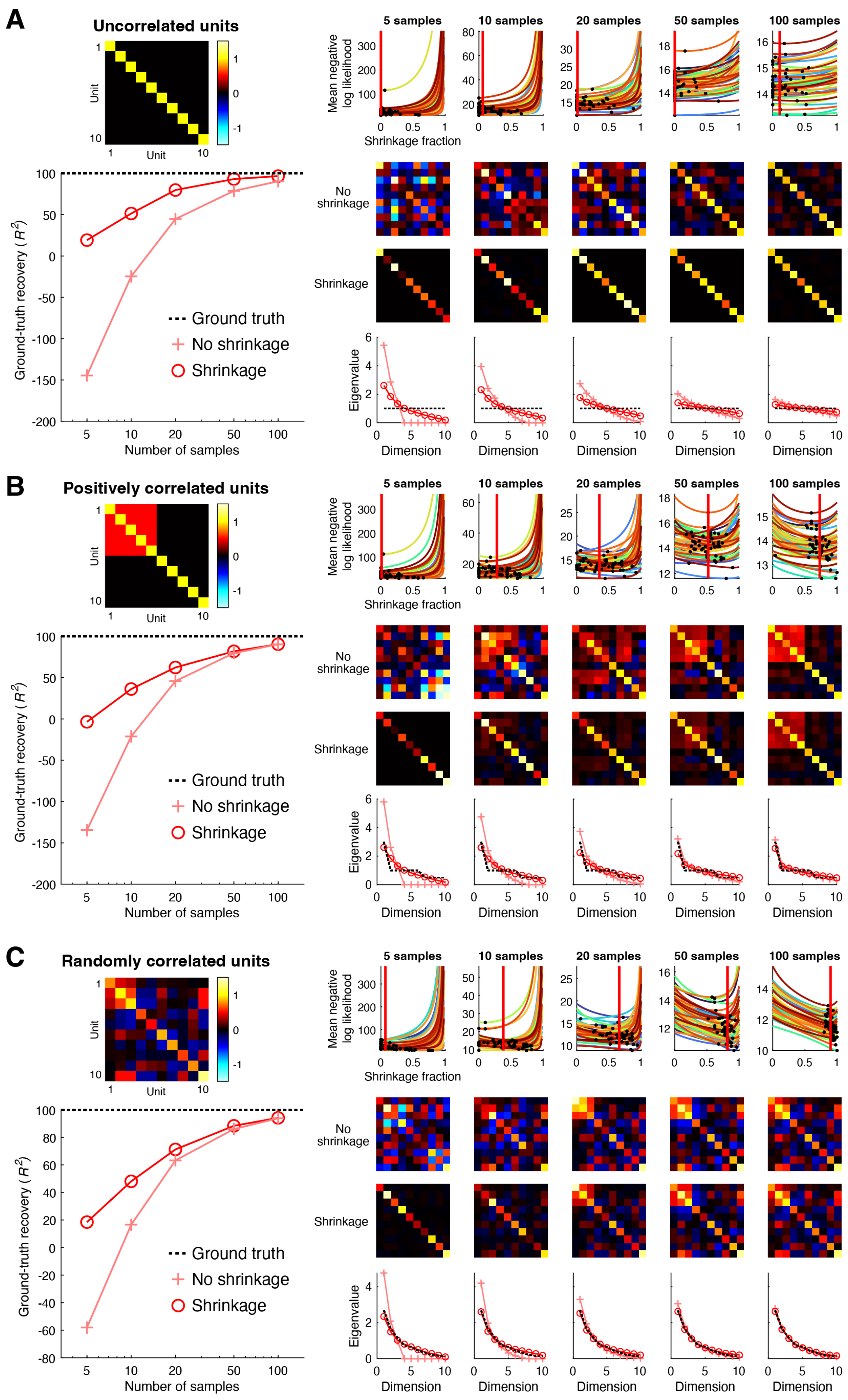

bioRxiv preprint doi: https://doi.org/10.1101/2024.04.22.590510; this version posted August 22, 2024. The copyright holder for this preprint (which was not certified by peer review) is the author/funder, who has granted bioRxiv a license to display the preprint in perpetuity. It is made available under aCC-BY 4.0 International license.

Figure S6.1. Shrinkage-based covariance estimation. Here we show results of simulations that assess the performance of the shrinkage-based method we use for covariance estimation (code available at https://osf.io/yr3vx). Panels A–C depict three different scenarios. Each scenario involves 10 units whose responses are distributed according to a ground-truth multivariate Gaussian (whose covariance is shown at the upper left). We vary the number of samples (e.g., trials, conditions) drawn from the distribution, performing 50 simulations for each number of samples. In each simulation, we estimate covariance from the samples using two different methods. One method ('No shrinkage') is to simply compute the sample covariance with Bessel's correction. The second method ('Shrinkage') involves additionally shrinking the off-diagonal elements of the sample covariance, using cross-validation to determine the optimal shrinkage level. In each panel, the ground-truth covariance is shown at the upper left. Cross-validation results for different numbers of samples are shown at the upper right, where colored lines indicate different simulations, black dots indicate the minimum negative log likelihood achieved, and the vertical red line indicates the median selected shrinkage level across simulations. Below each cross-validation plot, covariance estimates from one simulation are shown (we choose the simulation in which the selected shrinkage level is closest to the median). At the bottom are plots of the eigenspectra (mean across simulations) produced by the two methods (red and pink lines) as well as the ground-truth eigenspectrum (black dotted line). Finally, the ground-truth recovery performance quantified using coefficient of determination (R^2) is shown at the lower left (mean across simulations).

A clear benefit of the bias induced by shrinkage can be seen in the eigenspectra of the covariance estimates (panels A–C, bottom right). Even though the sample covariance provides an unbiased estimate of covariance, it produces biased eigenspectra that are lower in dimensionality than the ground-truth eigenspectra (see steep fall-off of the eigenspectra in the case of 5 samples). In other words, the sample covariance tends to underestimate the true dimensionality of the data. Shrinkage, to an extent, alleviates this issue, as it increases dimensionality (eigenvalues become more spread out) and produces eigenspectra that more closely resemble the ground-truth eigenspectra. These results are consistent with prior results from the literature (see Figure 1 in (Schäfer and Strimmer, 2005)).
